# Knowledge and attitudes of university students toward pandemic influenza: a cross-sectional study from Turkey

**DOI:** 10.1186/1471-2458-10-413

**Published:** 2010-07-13

**Authors:** Hulya Akan, Yesim Gurol, Guldal Izbirak, Sukran Ozdatlı, Gulden Yilmaz, Ayca Vitrinel, Osman Hayran

**Affiliations:** 1 Department of Family Medicine, Yeditepe University Faculty of Medicine, İnönü Mahallesi, Kayışdağı Cad., 26 Ağustos Yerleşimi, 34755 Kadıköy - İstanbul, Turkey; 2 Department of Microbiology and Clinical Microbiology, Yeditepe University Faculty of Medicine, İnönü Mahallesi, Kayışdağı Cad., 26 Ağustos Yerleşimi, 34755 Kadıköy - İstanbul, Turkey; 3 Yeditepe University Faculty of Pharmacy, İnönü Mahallesi, Kayışdağı Cad., 26 Ağustos Yerleşimi, 34755 Kadıköy - İstanbul, Turkey; 4 Department of Pediatric Health and Diseases, Yeditepe University Faculty of Medicine, İnönü Mahallesi, Kayışdağı Cad., 26 Ağustos Yerleşimi, 34755 Kadıköy - İstanbul, Turkey; 5 Department of Public Health, Yeditepe University Faculty of Medicine, İnönü Mahallesi, Kayışdağı Cad., 26 Ağustos Yerleşimi, 34755 Kadıköy - İstanbul, Turkey

## Abstract

**Background:**

During an influenza pandemic, higher education institutions with large populations of young adults can become serious outbreak centers. Since outbreak management is essential to disease control, we aimed to examine university students' knowledge of and attitudes toward the pandemic influenza A/H1N1 and vaccination and other preventive measures.

**Methods:**

A cross-sectional study was conducted among 402 first year university students at Yeditepe University in Istanbul, Turkey between 1^st ^and 30^th ^of November 2009. Data regarding socio-demographic characteristics of the students, perceptions, level of knowledge and attitudes toward influenza pandemic and prevention measures were collected by means of a self-administered questionnaire. The questionnaire was distributed by the students affiliated with SANITAS, a university club of students in health related sciences.

**Results:**

25.1% (101/402) of the study group perceived their personal risk of influenza as "high", while 40.5% (163/402) perceived it as "moderate", 20.6% (107/402) viewed it as "low" and 7.7% (31/402) indicated that it was "unknown". The risk perception of males was significantly lower than that of females (p = 0.004) and the risk perception among the students of health sciences was significantly lower than that of students of other sciences (p = 0.037). Within the study group, 72.1% (290/402) indicated that their main information source regarding H1N1 was the mass media. Health sciences students tended to rely more on the internet as an information source than other students (p = 0.015). The vast majority (92.8%; 373/402) of those interviewed indicated that they would not be vaccinated. The major concerns regarding vaccination had to do with the safety and side effects of the vaccine. Most of the participants (343/402, 85.3%) were carrying out one of prevention measures and the vast majority believed that hand washing, face mask and quarantina were effective measures for prevention.

**Conclusion:**

The participants had enough knowledge about H1N1 pandemic about the disease although there were still gaps and confusions in some areas. In the future, when planning management strategies regarding pandemics or outbreaks in higher education institutions, new strategies should be developed to promote positive health behaviour among university students compatible with the international guidelines. Main information source is mass media, so it seems that new policies must be developed to attract attention of students to use different and more scientific-based information sources.

## Background

Novel strains of influenza, H5N1 in recent years [1,2] and now H1N1 swine influenza [3-5], have made pandemic planning a priority for health authorities since the behaviour of new mutations are unpredictable and may result in millions of death.

A worldwide response to emerging diseases coalesced in the World Health Organization (WHO) Global Outbreak Alert and response Network, established in 2000 [6]. Since then, WHO resource documents have guided the development of national pandemic influenza plans and rapid action where outbreaks have occurred [7]. The first confirmed case of influenza A/H1N1 was reported on 23 April 2009 in Mexico [8]. On 11 June 2009, WHO raised the level of influenza pandemic alert from phase 5 to phase 6 [9].

Turkey responded rapidly to the pandemic influenza alert. In Turkey, the first case was reported on 18 June 2009, the first school case was reported on 13 December 2009 and the first death was on 22 December 2009. As of 12 December 2009, according to Ministery of Health (MOH) estimates, there were approximately 3.637.630 cases and 56% of cases were between 5-24 yrs; there have been 507 deaths in Turkey. As a pandemic strategy the MOH coordinated related agencies including all health services, ordered vaccine and stockpiled enough antivirals. The MOH also initiated studies to increase public knowledge and awareness. The vaccination against influenza A/H1N1 which was initiated on 02.11.2009, was provided free http://www.grip.gov.tr.

Public compliance with government directives is as important as well-organized pandemic response. Failure to comply with government directives in emergency situations may put public health at risk. Public perceptions, beliefs and knowledge have a critical impact on the spread of pandemics. If people are provided with accurate and accesible information from health authorities about the disease and appropiate responses and are made aware that the consequences of not complying with those recommendations are likely to dire, they are more likely to comply with health directives, and in turn, play a key role in stemming spread of the spread of the pandemic [10-13].

Higher education institutions with their large concentrations of young people have the potential to become serious outbreak centers. This indirectly may have a negative effect in the community. In this context CDC published a guide for strategies to be carried out against pandemic, for instutions of higher education during 2009-2010 calendar year on 22.02.2010 [14].

So, in this study we aimed to examine university students' knowledge about and attitudes toward the pandemic influenza A/H1N1 and the vaccination against it and other preventative measures.

## Methods

### Context

This cross-sectional study was conducted at Yeditepe University, Istanbul, Turkey from 1- 30 November 2009. The timing of the study was just after the peak point of the H1N1 pandemic in Northern countries, including Turkey. As a pandemic strategy the MOH initiated studies to increase public awareness very early including providing information on the internet, educating school teachers and elementary school children, developing information centers to respond to questions from the public, publishing leaflets and brochures and airing television programmes consistent with guidelines of the WHO. The public information provided included guidance on hand washing, cough etiquette, face mask usage and general hygiene. It also described how the infection spreaded, need for isolation during the disease, defined people under high risk, provided information use of antivirals and encouraged people to undergo vaccination.

At Yeditepe University, Department of Infectious Diseases and Clinical Microbiology posted information on the university's website about the H1N1 pandemic. Also two seminars about the pandemic open to all students had been carried out.

On November 24^th ^2009, the MOH initiated vaccinations for those in the 2 to 24 year old age group, which had been designated by the Ministry as the fourth prioritized population group.

### Participants

The target population was first year university students at Yeditepe University. Yeditepe University is a foundation university located on the Asian side of Istanbul. In 2009, 13000 undergraduate students were registered in 11 colleges and 61 departments and a total of 1950 first year students were registered. The minimum sample size to represent this population at 95% confidence level was calculated to be 384. We selected our study sample by employing a systematic sampling method. One in every four students in student enrollment lists was selected for the study. The number of students selected for the sample was 486. We collected data from 402 of this group (participation rate: 402/486), because some of the students were not available during the study period and some did not want to participate in the study.

### Data collection

Data were collected by self-administered questionnaire. The questionnaire was prepared by the study group based on relevant literature and included open-ended, multiple choice and Likert Scale-type questions regarding the socio-demographic caharacteristics of the students, levels of knowledge and attitudes toward H1N1 influenza pandemic and prevention measures. Survey questionnaires were pilot tested with eight students in an English prep class at Yeditepe University on 22 October 2009. Questionnaires distributed to the study group in their classrooms by SANITAS members. SANITAS is a student club at Yeditepe University whose members are volunteer students from Faculties of Medicine, Pharmacy, Dentistry and Health Sciences. The overall study was approved by Clinical Research Evaluation Committee of Medical Faculty. Every student who filled in the questionnaire was informed of the purpose of the out over the fallowing two days.

### Statistics

The collected data was analyzed by SPSS v.11.5. Frequencies and percentages were computed for descriptive purposes and X^2 ^test was administered to test significance. Significance was considered when p < 0.05.

## Results

Among the 402 university students who answered the questionnaire, 231/402 (42.5%) were female and 171/402 (57.5%) were male, 102/402 (25.5%) were enrolled in health-related faculties including medicine, pharmacy, health sciences and dentistry, and mean age was 20.58 ± 2.90.

Table 1 presents the participants' perceptions of infection self-risk broken down according to gender and field of study. As evidenced, 25.1% (101/402) of the participants viewed the risk of becoming infected with swine influenza as high, 40.5% (163/402) believed the risk was moderate, 26.6% (107/402) believed it was low and 7.7% (31/402) indicated that it was unknown. The self-risk perceptions of the participants were significantly different according to gender and field of study; the risk perception of males was lower than females (p = 0.004) and the risk perception of the students in health sciences was lower than the students of other areas (p = 0.037).

**Table 1:** Risk perceptions of the students in the study group according to gender and field of study

	High	Moderate	Low	Unknown	Total
	n ( % )	n ( % )	n ( % )	n ( % )	n ( % )
***Gender***					
Male	37 (21.6)	59 (34.5)	61 (35.7)	14 (8.2)	171 (100.0)
Female	64 (27.7)	104 (45.0)	46 (19.9)	17 (7.4)	231 (100.0)
Total	101 (25.1)	163 (40.5)	107 (26.6)	31 (7.7)	402 (100.0)
		X^2 ^= 13.377	p = 0.004		
Field of study					
Health sciences	34 (33.3)	37 (36.3)	20 (19.6)	11 (10.8)	102 (100.0)
Others	67 (22.3)	126 (42.0)	87 (29.0)	20 (6.7)	300 (100.0)
Total	101 (25.1)	163 (40.5)	107 (26.6)	31 (7.7)	402 (100.0)
		X^2 ^= 8.487	p = 0.037		

The self-risk perception was not significantly different according to gender among health sciences students (p = 0.059), but was lower for males than females among the students of other faculties (p = 0.028).

Table 2 analyses participants' knowledge of and attitudes toward swine influenza based on Likert scale questions. The vast majority of the participants were aware of swine influenza the global pandemic and the outbreak in Turkey. A full 82.1% (329/402) of the participants believed that there was also a seasonal influenza outbreak in Turkey, although at the time of the study there were only few confirmed cases of seasonal influenza. 62.4% (250/402) of the participants believed that swine influenza was a modified form of seasonal influenza. Most knew that swine influenza was transmitted from human to human (84.5%, 339/402). 53.8% (224/402) believed that it was fatal.

**Table 2:** Likert scale questions of knowledge about and attitudes toward swine influenza of the participants

	I disagree	I don't know	I agree	Missing
	(n, %)	(n, %)	(n, %)	(n,%)
There is a swine influenza outbreak in the world currently	47(11.7)	16 (4.0)	324 (80.6)	15 (3.7)
There is a seasonal influenza outbreak in the world	47 (11.7)	50 (12.5)	304 (75.6)	1 (0.2)
Swine influenza is a varied form of seasonal influenza	79 (19.7)	71 (17.6)	250 (62.2)	2 (0.5)
Swine influenza could cause fatalities	122(30.4)	56 (13.9)	224(55.7)	0 (0.0)
Swine influenza transmits from human to human	35 (8.7)	27 (6.7)	339 (84.4)	1 (0.2)
There is a swine influenza outbreak in our country currently	42 (10.4)	30 (7.5)	329 (81.9)	1 (0.2)
Swine influenza isn't more serious than seasonal influenza	125 (31.1)	73 (18.3)	202 (50.1)	2 (0.5)
There is a seasonal influenza outbreak in our country	77 (19.2)	63 (15.7)	261 (64.9)	1 (0.2)
If I catch swine influenza, I will have the mild symptoms of the disease	64 (16.0)	160 (39.8)	178(44.2)	0 (0.0)
Healthy people do not get swine influenza that easily	120 (29.9)	56 (13.9)	225 (56.0)	1 (0.2)
I have enough knowledge about swine influenza	86 (21.4)	75 (18.7)	238 (59.2)	3 (0.7)
If I take enough precaution I won't be infected even I am not vaccinated	78 (19.2)	62 (15.4)	262 (65.2)	1 (0.2)
Swine influenza antiviral treatment cures the disease completely	205 (50.9)	135 (33.6)	60 (15.0)	2 (0.5)
Everyone infected with swine influenza is required to use antiviral drug	142 (35.3)	120 (29.9)	138 (34.3)	2 (0.5)
Anyone who comes to contact with someone infected with swine influenza is required to use antiviral drug	169 (42.0)	109 (27.1)	123 (30.5)	2 (0.5)
Swine influenza vaccine need only be provided to those who in high risk groups	132 (32.8)	92 (22.9)	178 (44.3)	0 (0.0)
Swine influenza vaccine has few side effects	195(48.5)	146 (36.3)	61 (15.2)	0 (0.0)
Seasonal influenza vaccine keeps also from swine influenza	204 (50.7)	144 (35.8)	52 (13.0)	2 (0.5)
I think everyone should be given swine influenza vaccine	260 (64.7)	78 (19.4)	60 (14.9)	4 (1.0)
Not enough information has been provided about swine influenza	111 (27.6)	69 (17.2)	221 (55.0)	1 (0.2)
Swine influenza vaccine and seasonal influenza vaccine must be provided together	159 (39.6)	162 (40.3)	81 (20.1)	0 (0.0)
The vaccine will stop the outbreak	225(56.0)	110 (27.4)	67(16.6)	0 (0.0)

50.5% (205/402) of the participants thought that swine influenza was less serious than seasonal influenza. 55.7% (225/402) of the participants believed that healthy people cannot catch swine influenza so easily and 65.3% (262/402) thought that if they took enough precautions they wouldn't be infected even they had not been vaccinated. More than half of the participants believed that they had sufficient knowledge about swine influenza (238/402, 59.7%). Almost one-third of the participants had no idea about antiviral drugs and antiviral treatment (Table 2).

More than half of the study participants (25/402, 54%) believed that the vaccine could stop the swine influenza outbreak, 65.3% (195/402) stated that the vaccine need not be provided to entire population and 48.5% believed that the vaccine had side effects. 59.6% (221/402) of the participants thought that insufficient information had been provided regarding the vaccine against swine influenza (table 2).

The vast majority of participants (92.8%, 373/402) stated that they were unwilling to be vaccinated against influenza A/H1N1 while only 7.2% (29/402) of participants said they would be. The percentage of participants agreeing to the vaccination did not statistically differ according to gender but there was significant difference according to field of study. A full 13.5% (14/102) students in health related sciences agreed to be vaccinated, while only 5% (15/300) of other students did (p = 0.003) (table 3).

**Table 3:** Attitudes of the students in the study group toward vaccination according to gender and field of study

	Accept vaccination	Against vaccination	Total
	n (%)	n (%)	n (%)
***Gender***			
Men	13 (7.6)	158 (92.4)	171 (100.0)
Women	16 (6.9)	215 (93.1)	231 (100.0)
Total	29 (7.2)	373 (92.8)	402 (100.0)
	X^2 ^= 0.67	P > 0.05	
***Field of study***			
Health sciences	14 (13.7)	88 (86.3)	102 (100.0)
Other than health sciences	15 (5.0)	285 (95.0)	300 (100.0)
Total	29 (7.2)	373 (92.8)	402 (100.0)
	X^2 ^= 8.658	p = 0.003	

The participants' explanations for not wanting to be vaccinated are presented in Table 4. Their main concerns related to the vaccination's safety.

**Table 4:** The reasons for saying "No" to vaccination in the study group

REASON	Number (n)	Percent (%)
It is not safe	106	26.4
I don't trust it	86	21.3
I don't need it, since I am not in a risk group	62	15.3
Ineffective	30	7.5
It is too late, I will wait for the vaccines produced in USA	17	4.2
I don't want to be a guinea pig	9	2.2
Various reasons	26	6.3
I don't know	21	5.8
Missing	44	11.0
*TOTAL*	*402*	*100*

"What do you do for protection" was the survey's one of open-ended questions. A total of 370 of the 402 participants answered the question. Only a 14.7% (59/402) of those who answered stated that they did nothing for protection. The rest described various precautions. 53.5% (215/402) students took protective measures in their hygiene (including hand washing and generalcleanliness); 3.7% (15/402) took precautions in their for diet; 11.2% (45/402) did so both in hygiene and diet. 5.0% (20/402) limited their contact with others; 1.7% (7/402) students took vitamins, 1.5% (6/402) monitored their immunity and 0.7% (3/402) followed the suggestions of the MOH.

Table 5 analyses perceptions of the effectiveness of preventive measures regarding the transmission of swine influenza. Most participants believed, hand washing was effective (304/402, 75.9%). A slightly smaller portion believed wearing face mask (262/402, 65.3%) and quarantine (277/402, 69.4%) were effective. Only 39.1% (155/402) believed swine influenza vaccine was effective while a full 46.9% (188/402) believed herbs were effective for prevention.

**Table 5:** Distribution of the anwers to the effectiveness of the preventive measures in the study group

	Very effective	Moderatelyeffective	Low effective	Ineffective	I don't know	Missing
	n (%)	n (%)	n (%)	n (%)	n (%)	n (%)
Herbs	76 (19.0)	112 (27.9)	78 (19.5)	52 (13.0)	83 (20.7)	1 (0.2)
Seasonal influenza vaccine	21 (5.2)	89 (22.2)	110 (27.5)	99 (24.8)	81 (20.2)	2 (0.5)
Antivirals	50 (12.7)	105 (26.7)	68 (17.3)	37 (9.4)	133 (33.8)	9 (2.2)
Swine influenza vaccine	62 (15.6)	93 (23.5)	56 (14.1)	60 (15.2)	125 (31.6)	6 (1.5)
Hand washing	183 (45.6)	121 (30.2)	58 (14.5)	14 (3.5)	25 (6.2)	1 (0.2)
Face mask	130 (32.4)	132 (32.9)	72 (18.0)	33 (8.2)	34 (8.5)	1 (0.2)
Quarantine	200 (50.1)	77 (19.3)	44 (11.0)	29 (7.3)	49 (12.3)	3 (0.7)

Table 6 addresses the distribution of information sources about pandemic influenza according to participants' gender and field of study. The participants' main source about the H1N1 pandemic was the media (72.1%, 290/402) while 19.9% (80/402) came from the internet and 8.0% (32/402) from the health personnel consultation. There was no significant difference between the information sources of males and females, while internet use as a source of information was significantly higher among students of health sciences than students of other sciences (p = 0.015).

**Table 6:** The distribution of information sources about H1N1 according to gender and field of study in the study group

	Media	Internet	Health personnel	Total
	n ( % )	n ( % )	n ( % )	n ( %)
***Gender***				
Male	123 (71.9)	32 (18.7)	16 (9.4)	171 (100.0)
Female	167 (72.3)	48 (20.8)	16 (6.9)	231 (100.0)
Total	290 (72.1)	80 (19.9)	32 (8.0)	402 (100.0)
	X^2 ^= 0.942	P > 0.05		
***Field of study***				
Health sciences	63 (61.8)	30 (29.4)	9 (8.8)	102 (100.0)
Other than health sciences	227 (75.7)	50 (16.7)	23 (7.7)	300 (100.0)
Total	290 (72.1)	80 (19.9)	32 (8.0)	402 (100.0)
	X^2 ^= 8.380	p = 0.015		

## Discussion

Since higher education institutions have potential of becoming serious outbreak centers during a pandemic, the knowledge and attitudes of the university students toward a pandemic are important for compliance to the directives of health authorities. In our study group, most of the university students knew that there was H1N1 outbreak in the world and in our country and believed that they had sufficient knowoledge about the subject, but had less knowledge about the availability and effectiveness of antiviral drugs. Also, it seems that the terms of seasonal influenza and pandemic influenza were confused with each other. Most of the students thought that swine influenza was a modified form of seasonal influenza and that there was also a seasonal influenza outbreak in Turkey although only few such cases has been identified before or during the study period. Most of them knew that H1N1 influenza virus transmits from human to human. Although more than half of them believed that swine influenza could cause fatalities, most of them thought that if they caught the infection, the disease progress would have been mild.

Mass media was the most important information source consistent with previous studies regarding influenza pandemics [15-17], although students of health sciences relied more heavily on the internet. In their study which was about perceptions of a potential avian influenza pandemic in general public in 2007 , Kristiansen et al found that the majority of respondents had received information from the mass media (television %95, new papers 85%) [15]. Also, in the study of Kamate et al which was carried on during H1N1 pandemic, the major source of information was television (38.6%) [16]. In their statewide survey, Paek et al concluded that building close and amiable relationships with the media might also have been critical so that at the time of pandemic crisis, the media could play a role in disseminating information, not fear [17]. In fact we expected more internet use and consulting to health professionals as information source in this special group.

The self-risk perception was not high in most of the students. Risk perception was higher in females than males in the whole group, but this difference was not observed among the students in health sciences. It is well-known that there are gender differences in the context of risk perception. Perceptions of environmental health risks are much higher in female, for example, although underlying dynamics are not well understood [18,19]. Education in health related sciences may have abolished the gender difference in personal risk perception.

There are variable results related to the risk perception of swine influenza in different studies [20-22]. In their study Rubin et al found that 21% of their study which was conducted just at the beginning of the pandemic, population had high anxiety about swine influenza [20]. Barr et al found that 45.5% of their study population was very or extremely concerned that they or their family would have been affected, although at the time of this study avian influenza pandemic was expected [21]. Lau et al found that 10% of the participants considered themselves to have high or very high chance of contracting influenza A/H1N1 in the pre-pandemic period[22]. It is difficult to compare the results, since these studies have been conducted before or at the very beginning of the pandemic, also in diffrent countries and their cohort was general public.

Risk perception and compliance with preventive measures may have changed during the pandemic period. In a very recent study conducted during the peak period of the pandemic in Australia, Van et al showed that perception of susceptibility of university students and staff in the University of New South Wales significantly decreased with the decline of laboratory confirmed cases [23]. In their study, whilst 60.4% believed that it was serious, 64.2% reported either"no anxiety" or "disinterest" at the beginning of the study and towards the end of the survey period and the end of the winter, the percentage reporting "no anxiety" increased and the proportion of participants who believed pandemic was serious decreased. It was not possible to evaluate any change related with pandemic period in attitudes with this study. We expected higher self-risk perception since the study was conducted at the peak point of the outbreak. But, most of the students believed that healthy people do not get swine influenza very easily and if they had taken enough precautions they would not be infected even they hadn't been vaccinated which might have effected their risk perceptions.

Students in the study group believed that hand washing, wearing face mask and quarantine were the most effective measures regarding prevention and all but 15.9% were engaging in at least one preventative measure at the time of the study. In Van et al study, 61.8% of students had not undertaken any specific health behaviour; this percetage was quite high when compared with our results [23]. During the pandemic, the WHO and MOH strongly recommended good hygiene as means of limiting the spread of swine influenza. There is limited evidence, however, regarding the impact of wearing face mask, engaging in appropiate "cough etiquette" or hand washing[24]. While studies and recommendations support the proposition that non-pharmaceutical interventions lower the risk of respiratory infection , they do not point to a reduction in the susceptibility of population[25-29]. It appears that while hygienic behaviour is accepted by general population; compliance may be affected by many factors including the timing of outbreak, risk perception, responsibility for others, personal habits and sociodemographic factors [17,20,21,30].

Literature holds that the only way to prevent an influenza wave is an effective vaccination program and it is important to increase and coordinate preventive activities to slow virus transmission and to provide enough time for the preparation and distribution of the vaccine[3,4]. Kilbourne stated that "no amount of hand washing, public education or gauze masks will do the trick and the keystone of influenza prevention is vaccination"[31]. In the previous studies, there were differences regarding public support to the vaccine of influenza A/H1N1 [21,22,32,33]. In their study, Van et al found that 60% of university students and staff were willing to be vaccinated in pre-pandemic period [23]. But they also stated in their discussion, when the pandemic vaccine had been available after their study was ended, vaccination rate was 14% between 8-64 yrs according to Australian National Survey [34]. Although it was diffucult to compare because of the difference in timing and cohort of these studies, the percentage of those willing to be vaccinated in our study group was -7.2%- much lower. Although participants attending health related science were significantly more willing to be vaccinated, still when compared to the other studies the percentage was quite low. Similarly, the participants in our study expressed a negative attitude was observed toward influenza A/H1N1 vaccine and it was perceived the least effective prevention measure by the participants. Surprisingly, a higher proportion believed that herbal remedies were effective. The main reasons provided by our participants for not agreeing to take the vaccine were that it was new, that it might not be effective or safe and a perception that it had many side effects. Also, they thought that they had not been given adequate information about A/H1N1 vaccine. In their study, Sypsa et al. observed that the percentage of people that would probably not/definitely not accept the vaccine increased from 47.1% to 63.1% between 35^th ^to 44^th ^week and for the majority of the respondents the main reason for refusing vaccination was the belief the vaccine might have not been be safe [32]. Lau et al expressed that acceptibility of the A/H1N1 vaccine was highly sensitive to cost and also stated that people worlwide were curious about its effectiveness and safety and unless scientific evidence was available, the rate of vaccination in the general populations would be low [22]. Cost is not a factor in Turkey since the vaccine is free and its distribution is managed by the MOH. Our participants were young and healthy and their risk perceptions were at the middle level and they believed that if they caught the swine influenza they would experience mild symptoms of the disease. These factors may have affected their attitudes toward the vaccination against H1N1, although it is known that younger populations are much more susceptible than the elderly [31]. But when we examine their reasons not to be vaccinated, it is seen that safety and effectiveness and lack of information are the major concerns consistent with other studies. At the time of the study there was much coverage in the media about the safety and effectiveness of the vaccine which may have effected our study population. In the light of the negative attitude toward the H1N1 vaccination, it is apparent that public information and outreach is as important to meet vaccination targets as the availability of medication and well-organized public health systems.

There are limitations of our study. These include the survey restricted to the first year university students of Yeditepe University, so it does not reflect Turkish population as a whole and also does not represent whole Turkish university students. The participants were quite homogenous as a group so it is impossible to compare knowledge about and attitudes toward swine influenza regarding sociodemographic context.

## Conclusions

Based on the results of our study, the participants were aware of H1N1 pandemic and had enough knowledge about the subject although there were still gaps and confusions in some points. Risk perceptions of students are low to moderate and will remain so unless a change in the characteristic of pandemic status occur. Non-medical prevention measures are well accepted and have been carried on by most of the participants. But there was a negative attitude toward influenza A/H1N1 vaccine despite the recommendations of the MOH.

We conclude that unless the safety and efficacy of influenza A/H1N1 vaccine have been proven, this negative attitude and resistance toward vaccination against swine influneza will continue. In the future, when planning management strategies regarding pandemics or outbreaks in higher education institutions, new strategies should be developed to promote positive health behaviour among university students compatible with the international guidelines. Main information source is mass media, so it seems that new policies must be developed to attract attention of students to use different and more scientific-based information sources.

## Competing interests

The authors declare that they have no competing interests.

## Authors' contributions

HA carried out the design and coordination of the study and data entry and analysis. HA also participated in the sequence alignment and drafted the manuscript. YG carried out the data entry and analysis and in the sequence alignment and drafted the manuscript. GI carried out data entry and analysis and in the sequence alignment and drafted the manuscript SO carried out the study and also conducted data entry GY participated in its design and coordination and helped to draft the manuscript. She was also a scientific consultant to the study. AV helped to draft the manuscript. OH designed the methodology of the study and performed the statistical analysis. OH also participated in its design and coordination and helped to draft the manuscript. All authors read and approved the final manuscript.

## Pre-publication history

The pre-publication history for this paper can be accessed here:

http://www.biomedcentral.com/1471-2458/10/413/prepub
